# Case Report: Pulmonary alveolar proteinosis and fibrosis associated with indium-tin-oxide exposure

**DOI:** 10.3389/fmed.2026.1856204

**Published:** 2026-05-18

**Authors:** Xinyao Li, Chuan Shi, Qiaoling Chen, Jiapeng Zhao, Suxia Wang, Yunhong Yin, Ruie Feng, Xinlun Tian, Kai-Feng Xu

**Affiliations:** 1 Department of Pulmonary and Critical Care Medicine, Peking Union Medical College Hospital, Chinese Academy of Medical Sciences and Peking Union Medical College, Beijing, China; 2 Department of Pathology, Peking Union Medical College Hospital, Chinese Academy of Medical Sciences and Peking Union Medical College, Beijing, China; 3 Laboratory of Electron Microscopy, Pathology Center, Peking University First Hospital, Beijing, China; 4 Department of Pulmonary and Critical Care Medicine, Qilu Hospital of Shandong University, Jinan, China; 5 State Key Laboratory of Complex Severe and Rare Diseases, Peking Union Medical College Hospital, Chinese Academy of Medical Sciences and Peking Union Medical College, Beijing, China

**Keywords:** indium tin oxide, occupational diseases, pulmonary alveolar proteinosis, pulmonary fibrosis, whole-lung lavage

## Abstract

**Background:**

Pulmonary alveolar proteinosis (PAP) is a rare respiratory disorder characterized by the excessive accumulation of surfactant material on alveolar surfaces and the dysfunction of alveolar macrophages. Indium-tin-oxide (ITO), a substance utilized in liquid crystal displays and solar panels, could be a potential cause of secondary PAP. Here, we report a rare case of interstitial lung disease in a solar panel processing worker with ITO exposure, which was pathologically confirmed as PAP and then progressed to pulmonary fibrosis.

**Case presentation:**

A 35-year-old male was admitted with a symptom of dysponea. Chest computed tomography revealed diffuse, bilateral ground-glass opacities and interlobular septal thickening in both lungs. Transbronchial lung biopsy was PAS-positive, confirming the diagnosis of PAP. The indium levels in the patient’s bronchoalveolar lavage fluid and plasma were far above the normal range. He underwent whole-lung lavage, but approximately 4 months later, he still developed progressive pulmonary fibrosis. Nintedanib showed no significant effect in slowing the decline of his lung function.

**Conclusion:**

When a patient has a definite history of indium exposure, the diagnosis of PAP should be considered. Workers need to wear protective measures daily, as lung diseases caused by ITO exposure are associated with a poor prognosis.

## Introduction

Pulmonary alveolar proteinosis (PAP) is a rare respiratory disorder characterized by the excessive accumulation of surfactant material on alveolar surfaces and the dysfunction of alveolar macrophages (1). PAP can be further classified into primary, secondary, and congenital PAP based on its pathogenesis. Secondary PAP is often caused by conditions such as immunosuppression, chronic immune diseases, the inhalation of toxic substances, or hematologic malignancies that impair alveolar macrophage function (2). Inhalational toxins currently recognized to cause secondary PAP include mainly silica (3) and aluminum alloys (4). In addition to these metals, indium, a soft, silver-white metal, which has been widely used in electronic device manufacturing since the 1990s (5), could be a potential cause of secondary PAP (6).

Common compounds of indium include indium oxide, indium-tin-oxide (ITO), indium hydroxide, indium chloride and indium arsenide (5, 7, 8). Among these, ITO is extensively utilized in products such as liquid crystal displays, flat-panel displays, and solar cells due to its unique transparency. Pulmonary diseases caused by ITO have only been reported in the last 20 years and remain relatively rare. Here, we report a case of interstitial lung disease in a solar panel processing worker with ITO exposure, which was pathologically confirmed as PAP and then progressed to pulmonary fibrosis, providing a reference for future clinical studies on ITO exposure-related lung diseases.

## Case presentation

**Figure 1:**
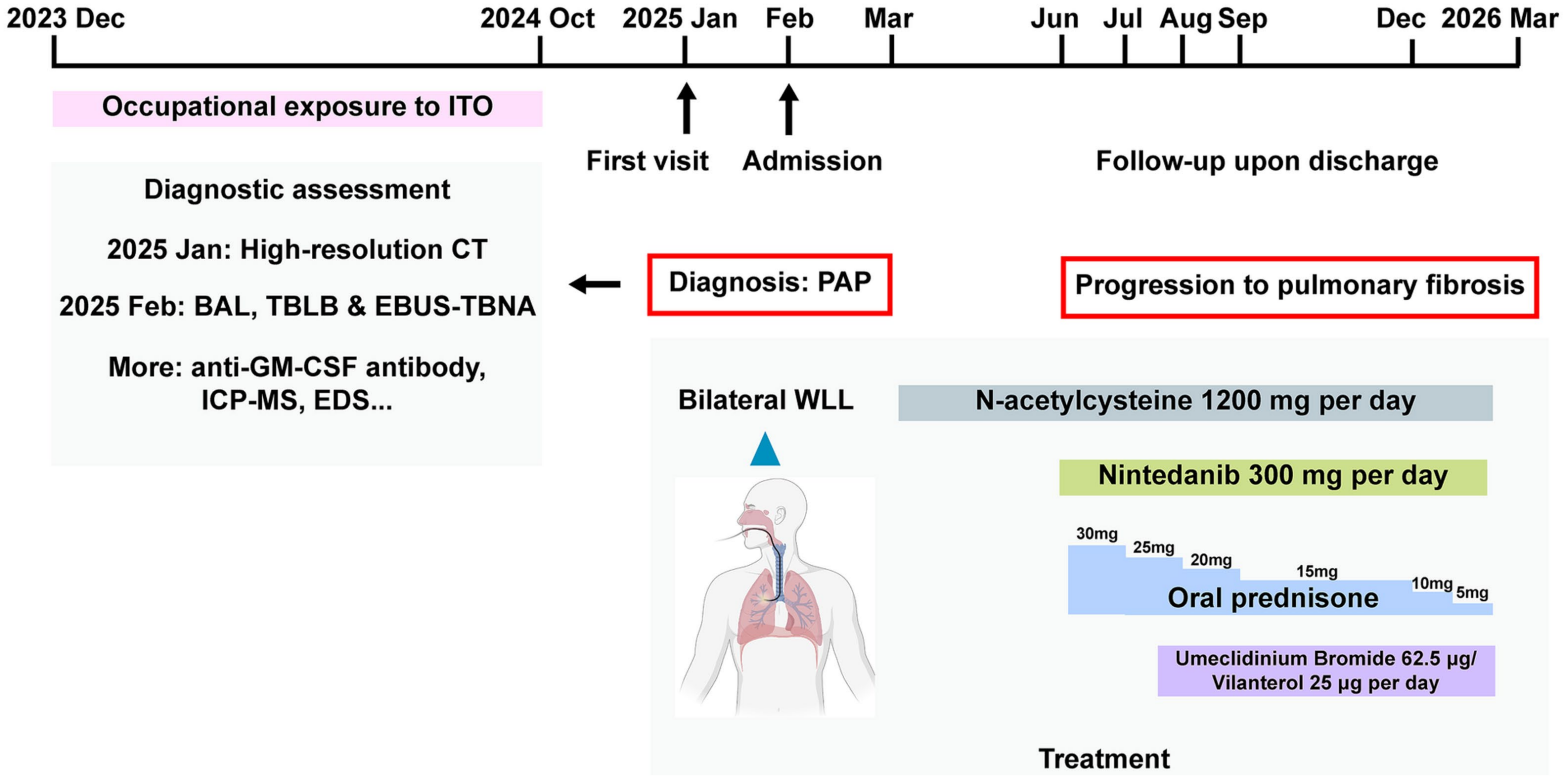
Graphical timeline of the present case. BAL, bronchoalveolar lavage; CT, computed tomography; EBUS-TBNA, endobronchial ultrasound-guided transbronchial needle aspiration; EDS, energy dispersive spectrometer; GM-CSF, granulocyte-macrophage colony-stimulating factor; ICP-MS, inductively coupled plasma-mass spectrometry; ITO, indium-tin-oxide; PAP, pulmonary alveolar proteinosis; TBLB, transbronchial lung biopsy; WLL, whole-lung lavage.

A 35-year-old male patient was referred to the Peking Union Medical College Hospital in January 2025 for the evaluation of diffuse parenchymal lung disease. The clinical timeline is summarized in Figure 1. He had been employed since December 2023 at an electronics factory. His job involved recovering ITO components from solar panels. He primarily used an electric hammer to separate the ITO film from the solar panels, a process that generates a large amount of dust. The patient worked in a confined environment with no windows or ventilation openings, except for a single suction duct that collects the dust for recycling. He works six days a week, ten hours a day. The patient reported that he wore protective suit, protective gloves, and either an N95 mask or a surgical mask, which he changed every 1–2 days. However, even when wearing a mask, he still inhaled a significant amount of dust into his mouth, nose. Prior to his current occupation, the patient had worked underground in a coal mine for six years from 2011 to 2017, during which period he was to dust and coal. He had no smoking or any medical history. In October 2024, the patient developed exertional dyspnea, which occurred after walking on level ground, climbing only a single level of stairs, or lifting heavy objects. His daily activities were limited, and he experienced intermittent coughing with small amounts of white sputum.

**Figure 2:**
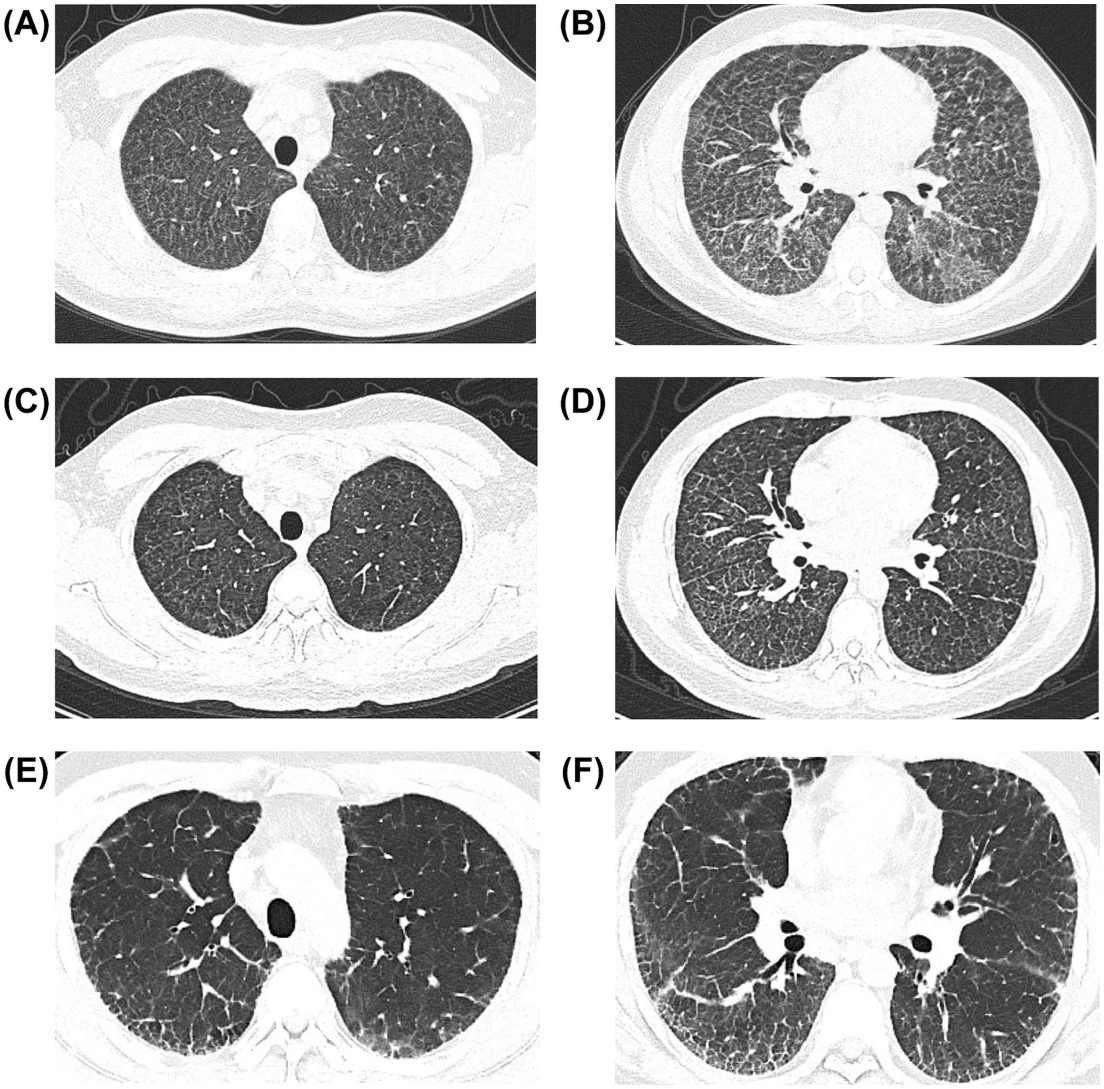
High-resolution computed tomography (HRCT) scans showing ground-glass opacification and interlobular septal thickening. The patients were scanned upon hospitalization in February 2025 **(A,B)**. HRCT revealed reduced ground-glass opacification 40 days after whole-lung lavage **(C,D)**. HRCT at 10 months after whole-lung lavage revealed a continuous reduction in ground-glass opacification; however, the reticular pattern increased **(E,F)**.

Over the following month, the patient experienced progressive worsening of symptoms and developed dyspnea at rest by January 2025. Oxygen saturation ranged between 75 and 80% on room air. The patient visited a local hospital, where chest computed tomography (CT) revealed diffuse lung disease. He was referred to our hospital for further evaluation and treatment. Physical examination revealed a pulse of 111 beats/min, a respiratory rate of 22 breaths/min, a blood pressure of 114/73 mmHg, and an oxygen saturation of 88% on room air. No skin rash was observed, and there was no lower limb edema or digital clubbing. Cardiopulmonary examination revealed bilateral rough breath sounds, with no audible dry or wet rales. Heart rhythm was regular, and no pathological murmurs were heard over any valve areas. Laboratory investigations revealed several abnormalities related to the concentrations of the following: lactate dehydrogenase (LDH) 687 U/L (normal range <250 U/L), carcinoembryonic antigen (CEA) 6.4 ng/mL (normal range ≤5 ng/mL), cytokeratin fragment 19 (Cyfra21-1) 6.5 ng/mL (normal range ≤3.5 ng/mL), Krebs Von den Lungen-6 (KL-6) 3630 U/mL (normal range 105–401 U/mL), and rheumatoid factor (RF) 55.2 IU/mL (normal range 0–20 IU/mL). All antinuclear antibody (ANA) spectra were negative. Liver and renal function tests, complete blood tests were normal. Arterial blood gas analysis on room air revealed the following: pH 7.41, PaCO₂ 33 mmHg, PaO₂ 54 mmHg, arterial oxygen saturation (SaO₂) 86.2%, and alveolar–arterial oxygen gradient (A–a gradient) 59.2 mmHg. Pulmonary function tests revealed reduced diffusing capacity along with a mild restrictive ventilatory defect (Table 1). High-resolution computed tomography (HRCT) of the chest revealed diffuse, bilateral ground-glass opacities and interlobular septal thickening in both lungs (Figures 2A,B).

**Table 1:** Pulmonary function during 12 months of follow-up.

Variables	Before WLL	40 days post-WLL	4 months post-WLL	10 months post-WLL
FVC (L) (%pred)	3.11 (78)	3.18 (80)	2.76 (70)	2.47 (63)
FEV1 (L) (%pred)	2.63 (78)	2.66 (79)	2.26 (67.6)	2.11 (63)
FEV1%FVC (%)	84.63	83.71	82.09	85.33
DLCO (mmol·min^−1^ k Pa^−1^) (%pred)	2.65 (26.16)	3.79 (40)	4.33 (46)	4.24 (44)

The numbers in the parentheses represent the ratio of the actually measured value to the predicted value. FVC, forced vital capacity; FEV1, forced expiratory volume in one second; DLCO, diffusing capacity of the lung for carbon monoxide; WLL, whole-lung lavage.

## Diagnostic assessment

**Figure 3:**
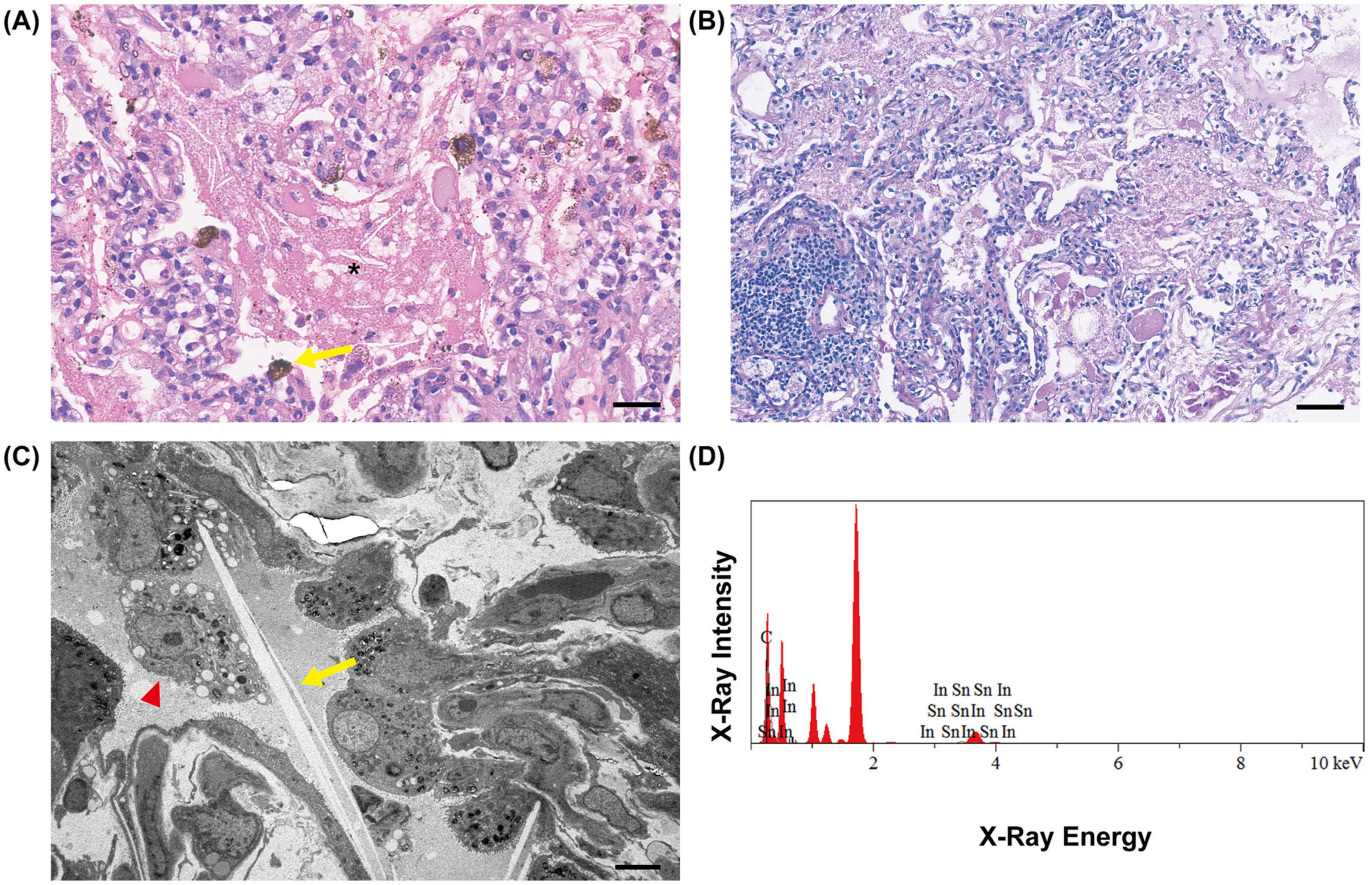
Histopathological sections of lung tissue. The alveolar filling with eosinophilic material (* label) is shown in **(A)** under hematoxylin and eosin staining, brown indium particles can also be identified (yellow arrow) (magnification × 40, bar = 50 μm). The tissue is positive under Periodic-acid-Schiff staining **(B)**, magnification × 20, bar = 100 μm. As shown in **(C)**, the macrophages contained foamy lipid droplets (red triangle) and needle-shaped cholesterol clefts (yellow arrow) (magnification × 2000, bar = 5 μm). **(D)** The result of energy dispersive X-ray analysis of particles. EDS detects the presence of different elements by directing electron beam to shoot the metallic particles deposited in the lung tissue. By measuring the wavelength and energy of the emitted X-rays from the particles and comparing them against the reference spectra of various elements in the database, indium (In) and tin (Sn) can be identified, indicating the patient’s exposure to ITO.

For diagnostic purposes, bronchoalveolar lavage (BAL), transbronchial lung biopsy (TBLB) and endobronchial ultrasound-guided transbronchial needle aspiration (EBUS-TBNA) were performed. After standing, the recovered lavage fluid appeared milky and turbid with white sediment. Histopathological examination revealed eosinophilic proteinaceous material within the alveoli (Figure 3A). Both periodic acid–Schiff (PAS) and diastase-resistant PAS (D-PAS) staining were positive (Figure 3B). Subsequent electron microscopy of lung tissue revealed colorless, slender, rod-shaped crystalline inclusions within the cytoplasm of alveolar macrophages, with some crystals secreted into the alveolar spaces (Figure 3C). The appearance of the BAL fluid (BALF), together with the pathological findings, was consistent with the diagnosis of PAP.

The serum concentration of anti–granulocyte–macrophage colony-stimulating factor (GM-CSF) antibodies was 1.3 μg/mL (normal range < 4.0 μg/mL), which ruled out autoimmune PAP. Based on the patient’s previous history of ITO exposure, inductively coupled plasma-mass spectrometry (ICP-MS) was performed on the patient’s BALF and plasma samples. The results revealed indium concentrations of 165.7 ng/mL in plasma and 202.1 ng/mL in BALF, which significantly exceeded the normal upper limit (<0.06 ng/mL). A portion of the lung tissue obtained by TBLB was used for energy dispersive spectrometer (EDS) analysis. EDS provided further confirmation of the existence of indium and tin (Figure 3D), proving the patient’s exposure to ITO. Based on the patient’s occupational history, clinical course, HRCT and pathologic findings, the patient was diagnosed with secondary pulmonary alveolar proteinosis. Furthermore, given that the patient had a history of indium exposure for more than six months and was diagnosed with pulmonary alveolar proteinosis, the patient also met the diagnostic criteria for occupational poisoning of indium compound (9).

The patient was admitted to our hospital in February 2025 for further treatment. He underwent whole-lung lavage (WLL) under general anesthesia. A total of 11,300 mL of normal saline was instilled into the left lung, and 11,000 mL was instilled into the right lung. During the procedure, the recovered fluid gradually changed from yellow and turbid to clear and transparent. Following WLL, there were improvements in symptoms (mMRC grade 2), oxygen saturation (93 ~ 95% on room air), imaging findings (Figures 2C,D), and diffusing capacity (Table 1).

However, the patient’s symptoms worsened over time. He was prescribed N-acetylcysteine (1,200 mg/d) 1 month after WLL, with additional oral steroids (initial dose prednisone 30 mg/d) and nintedanib (300 mg/d) 4 months after WLL. Ten months after WLL, the patient was stable in terms of activity tolerance but experienced further deterioration of lung function (Table 1). Repeated HRCT revealed subpleural fibrosis (Figures 2E,F). One year after WLL, the patient’s plasma was again submitted for ICP-MS analysis, which revealed an indium concentration of 7.6 ng/mL. Due to persistent limitations in daily activities, the patient has not yet returned to work.

## Discussion

We introduced a case of PAP in a patient with a significant occupational exposure history. His symptoms presented after 10 months of exposure to ITO and followed a rapidly progressive course. Ultimately, ICP-MS confirmed markedly elevated indium concentrations in his plasma and BALF, far exceeding the normal range. His initial histopathological pattern was pulmonary alveolar proteinosis diagnosed by BALF and EBUS-TBNA. To alleviate his symptoms and remove intrapulmonary indium, he underwent whole-lung lavage, which yielded suboptimal efficacy. Subsequently, pulmonary images showed progression toward pulmonary fibrosis, and nintedanib proved ineffective.

ITO is a sintered material with 90% indium-oxide (In_2_O_3_) and 10% tin-oxide (SnO_2_). In 2003, Homma first reported a case of indium-related lung disease (10). The patient developed subpleural honeycombing and diffuse ground-glass opacities after 3 years of exposure to ITO. Cases of indium-related lung disease were subsequently reported in the United States and China (11–13).

In 2012, Cummings et al. summarized all 10 published cases of indium-related lung disease (6). Among these, six patients presented with pulmonary fibrosis (latency: 4–13 years), while three manifested as PAP (latency: 1–2 years), and four patients with 0–18 pack-year smoking histories had emphysema. Notably, upon re-evaluation of the lung histopathology from these 10 patients, homogeneous granular eosinophilic material filling the alveolar spaces was observed in nine patients. Similarly, pulmonary fibrotic changes were identified in nine out of 10 cases, and cholesterol clefts were present in all 10 cases. A subsequent study evaluated 108 workers at an Japanese indium processing plant and assessed their symptoms, HRCT, and pulmonary function (14). The average blood indium concentration among these workers was 7.9 ± 4.3 ng/mL. Although none of the workers reported breathlessness on exertion, 23 (21%) had significant interstitial changes and 14 (13%) had significant emphysematous changes, and 40 (37%) had elevated KL-6 levels (>500 U/mL).

Research has demonstrated that compared with indium-oxide or tin-oxide alone, ITO has stronger proinflammatory effects and is uniquely capable of inducing a potent cytotoxic response in macrophages (15). However, the precise mechanisms underlying ITO-induced PAP remain incompletely understood. It was reported that phagocytic activity in BALF was significantly reduced in ITO-treated rats, suggesting that ITO deposition impaired the function of alveolar macrophages (16). Meanwhile, alveolar macrophage dysfunction is known to play a key role in the development of PAP (1). Rats with exposure to ITO demonstrated pathological changes consistent with PAP, with prolonged exposure leading to pulmonary fibrosis (17). Liu et al. demonstrated that exposure to ITO in rats activates the NF-κB/Nrf2 pathway, triggering oxidative stress and inflammatory responses (18), which contribute to the development of PAP. Radiographic data revealed that two patients with PAP subsequently developed fibrosis (6), consistent with our patient. These observations, together with previously reported cases, suggest that protein deposition and fibrosis represent sequential stages of disease progression rather than mutually exclusive changes. In our patient, the duration of indium exposure was approximately one year, and no changes in fibrosis were observed by histopathology, suggesting that the patient was still in the early stage of the disease at the time of biopsy and subsequently progressed to pulmonary fibrosis.

In terms of prognosis, patients experienced persistent progression after diagnosis. While one patient derived benefit from WLL, one experienced symptom resolution without specific treatment, the remaining eight cases eventually worsened over time despite therapy with WLL or steroids. Ultimately, two patients died, one from bilateral pneumothorax and another from respiratory failure. A longitudinal cohort study by Amata (19), which followed 84 workers for more than 9 years, revealed that blood indium levels exhibited a dose–response effect, with an estimated half-life of approximately 8.09 years. For workers with higher blood indium levels, emphysematous changes on HRCT continued to progress. Our patient’s initial plasma indium concentration was 165.7 ng/mL, which decreased to 7.6 ng/mL one year after WLL, indicating that WLL is effective in removing indium from the body. Although WLL may not provide long-term stabilization of the symptoms and lung function, it remained a necessary procedure to promptly remove deposited indium from the lungs and prevent more severe deterioration of the disease. Nakano et al. analyzed the incidence of lung cancer over 6 years in a multicenter cohort of indium-exposed workers (20). Among 220 exposed workers, 4 were diagnosed with lung cancer during follow-up. These findings suggest that annual imaging is necessary to detect any new pulmonary lesions and rule out the possibility of lung cancer.

Notably, previous research had reported that N-acetylcysteine (NAC) could effectively reduce the severity of protein deposition and fibrosis in the ITO-exposed group (18). The patient had been given a 10-month course of NAC therapy. No significant improvement in efficacy was observed, which may be attributable to the fact that NAC was administered as a therapeutic drug rather than as an intervention agent.

Our center is the first to have fully documented the disease course of a patient with ITO exposure progressing from PAP to pulmonary fibrosis. The rate of disease progression and the radiological changes observed in this patient provide valuable reference for future studies. Furthermore, although previous reports have suggested that WLL does not stabilize the disease, we emphasize the importance of WLL for the timely removal of indium from the body and for reducing ongoing indium exposure. However, our study has certain limitations. We have reported only one case of indium-related lung disease, and more patients are needed to confirm our conclusions. Additionally, given the high frequency of pulmonary fibrosis reported in indium lung disease, we should consider initiating antifibrotic therapy immediately after diagnosis to delay the decline in lung function.

## Conclusion

In summary, indium and its compounds are metals with pulmonary toxicity, and long-term exposure can lead to indium-related lung disease, which has a poor prognosis. Further studies are needed to clarify the mechanisms by which indium induces lung tissue injury and search for potential therapies. Given the risk of indium-related lung injuries, such as pulmonary fibrosis and pulmonary alveolar proteinosis, enterprises must prioritize environmental protection and monitor indium exposure levels, while workers are required to adhere to strict protective measures.

## Patient perspective

I was diagnosed with pulmonary alveolar proteinosis due to exposure to ITO. I eventually progressed to pulmonary fibrosis, which has caused certain difficulties in my daily life. I hope that in the future, there will be therapies to improve the symptoms of pulmonary fibrosis caused by ITO exposure, and that factories will provide more effective protective measures.

## Data Availability

The original contributions presented in the study are included in the article/supplementary material, further inquiries can be directed to the corresponding author.
